# *Aesculus hippocastanum* L. as a Stabilizer in Hemp Seed Oil Nanoemulsions for Potential Biomedical and Food Applications

**DOI:** 10.3390/ijms22020887

**Published:** 2021-01-17

**Authors:** Maciej Jarzębski, Wojciech Smułek, Przemysław Siejak, Ryszard Rezler, Jarosław Pawlicz, Tomasz Trzeciak, Małgorzata Jarzębska, Oliwia Majchrzak, Ewa Kaczorek, Pardis Kazemian, Marta Ponieważ-Pawlicz, Farahnaz Fathordoobady

**Affiliations:** 1 Department of Physics and Biophysics, Faculty of Food Science and Nutrition, Poznań University of Life Sciences, Wojska Polskiego 38/42, 60-637 Poznań, Poland; przemyslaw.siejak@up.poznan.pl (P.S.); ryszard.rezler@up.poznan.pl (R.R.); malgorzata.jarzebska@o2.pl (M.J.); 2 Institute of Chemical Technology and Engineering, Poznan University of Technology, Berdychowo 4, 60-695 Poznan, Poland; wojciech.smulek@put.poznan.pl (W.S.); oliwia.majchrzak@student.put.poznan.pl (O.M.); ewa.kaczorek@put.poznan.pl (E.K.); 3 Department of Orthopedics and Traumatology, Poznan University of Medical Sciences, 28 Czerwca 1956 135/147, 61-545 Poznań, Poland; jarekpawlicz@gmail.com (J.P.); trzeciak@orsk.ump.edu.pl (T.T.); 4 Faculty of Sciences, Department of Biology, University of British Columbia, 1103-6270 University Blvd Biological Sciences Building, Vancouver, BC V6T 1Z4, Canada; p.kazemian@alumni.ubc.ca; 5 Imaging Diagnostic Facility, St. Joseph Hospital, Krysiewicza 7/8, 61-825 Poznań, Poland; martaponiewaz@gmail.com; 6 Food, Nutrition and Health, University of British Columbia, 2205, East Mall, Vancouver, BC V6T 1Z4, Canada; farah.fathordoobady@ubc.ca

**Keywords:** horse chestnuts, *Cannabis sativa*, droplet size, dynamic light scattering, viscosity, emulsion, surface tension, light microscopy, hyaluronic acid

## Abstract

Nanoemulsion systems receive a significant amount of interest nowadays due to their promising potential in biomedicine and food technology. Using a two-step process, we produced a series of nanoemulsion systems with different concentrations of hemp seed oil (HSO) stabilized with *Aesculus hippocastanum* L. extract (AHE). Water and commercially-available low-concentrated hyaluronic acid (HA) were used as the liquid phase. Stability tests, including an emulsifying index (EI), and droplet size distribution tests performed by dynamic light scattering (DLS) proved the beneficial impact of AHE on the emulsion’s stability. After 7 days of storage, the EI for the water-based system was found to be around 100%, unlike the HA systems. The highest stability was achieved by an emulsion containing 5% HSO and 2 g/L AHE in water, as well as the HA solution. In order to obtain the detailed characteristics of the emulsions, UV-Vis and FTIR spectra were recorded, and the viscosity of the samples was determined. Finally, a visible microscopic analysis was used for the homogeneity evaluation of the samples, and was compared with the DLS results of the water system emulsion, which showed a desirable stability. The presented results demonstrate the possible use of oil emulsions based on a plant extract rich in saponins, such as AHE. Furthermore, it was found that the anti-inflammatory properties of AHE provide opportunities for the development of new emulsion formulations with health benefits.

## 1. Introduction

Nanoemulsions (NEs) are heterogeneous phases of nano-size droplets (50–500 nm) within a dispersant phase stabilized by emulsifiers or surfactants. They can be developed as both oil-in-water (O/W) and water-in-oil (W/O) formulations. A transparent appearance, excellent stability, and adjustable rheology are the distinctive properties of NEs, making them an effective alternative for applications in the cosmetic, food and pharmaceutical industries, as well as for drug delivery purposes [1,2]. Smart foods can be designed with NEs to incorporate with low aqueous solubilities. The application of NEs has also improved the digestibility of certain food formulations [3]. Drug administration via various delivery routes—including oral, transdermal, ocular, intravenous, and intranasal routes—can further be facilitated through NE formulations. In addition, the parenteral delivery of NEs has the potential to fulfil nutritional requirements, the delivery of vaccines, and controlled and targeted drug release [4].

Hemp seed oil from the seeds of *Cannabis sativa* L. is a potent source of ω-6 and ω-3 essential fatty acids, mainly linoleic acid (18:2 n-6) and α -linolenic acid (18:3 n-3), in the desired ratio (3:1) for human nutrition. The presence of δ-linolenic acid (18:3 n-6), stearidonic acid (18:4 n-3), and tocopherol, tocotrienols and carotenes adds more nutritional value to this oil [5,6,7]. The health benefits of hemp seed oil suggest several potential applications and specific nutritional formulations [8]. Besides its health and nutrition benefits, hemp seed oil can be more simply emulsified by emulsifiers due to its higher intersolubility with water compared to other vegetable oils [9]. Taking into account the fact that low surfactant-to-oil ratios are typically preferred [10], this will be in favor with the further reduction of using surfactants in HSO based nanoemulsion formulations.

The preparation of nanoemulsions with natural emulsifiers is effective, and it allows us to avoid the possible toxicity of synthetic surfactants. Among natural surface active compounds, saponins are plant-derived compounds with great health benefits, such as lowering cholesterol levels and tumor growth inhibition effects, which have gained commercial significance and expanded their applications due to their low cost and environmentally-friendly nature [11,12]. Different studies with various saponins groups have indicated the ability of these compounds to improve the emulsions’ characteristics. Saponins increase the stability of emulsions containing crude oil [13], pure hydrocarbons such as hexadecane, and natural oils including sunflower oil [14], rice bran oil [15] and olive oil [16]. It was also found that the presence of saponins led to a significant decrease in emulsion droplet size; however, the saponins’ impact on the droplets’ zeta potential was ambivalent [17,18]. When the emulsifying properties of saponins are considered, it should not be forgotten that their amphiphilic nature reduces the surface tension. The hydrophobic–hydrophilic structure of saponins also has a decisive influence on their biocidal activity, which is related to their interaction with biological membranes [19,20].

Saponins can be found in a number of plant tissues, including *Aesculus hippocastanum* L. (horse chestnuts) bark. Horse chestnut bark contains a compound called aescin, which has been shown to be effective for people with chronic venous insufficiency (CVI) through contracting veins and arteries. Furthermore, horse chestnut bark extract has protective effects against light radiation due to its absorption of wavelengths shorter than 370 nm and action as a free-radical scavenger [21,22]. Detailed spectroscopic studies of *Aesculus hippocastanum* L. bark extract (AHE) were presented in an earlier study [21]. Such properties make AHE a promising multifunctional additive for emulsion stabilization.

In this paper, we focused on the preparation of nanoemulsion systems based on HSO stabilized with AHE. For further potential food technology and biomedical applications, two liquid phases were used: water, and a commercially-available low-concentrated hyaluronic acid solution (Nebudose). The stability tests, such as emulsifying indices, were compared with the droplet size distribution (obtained by dynamic light scattering—DLS), zeta potential, and microscopic investigations. The viscosity and surface tension of the formulated emulsions were also analyzed here.

## 2. Results and Discussion

For the preliminary tests, a series of emulsion samples with different HSO and AHE concentrations were prepared (for details see p. 3.2.). As a liquid phase, water and a commercially-available HA solution (Nebudose) were used. Detailed sample descriptions are presented in Table 1.

### 2.1. Emulsion Stability Tests

**Figure 1:**
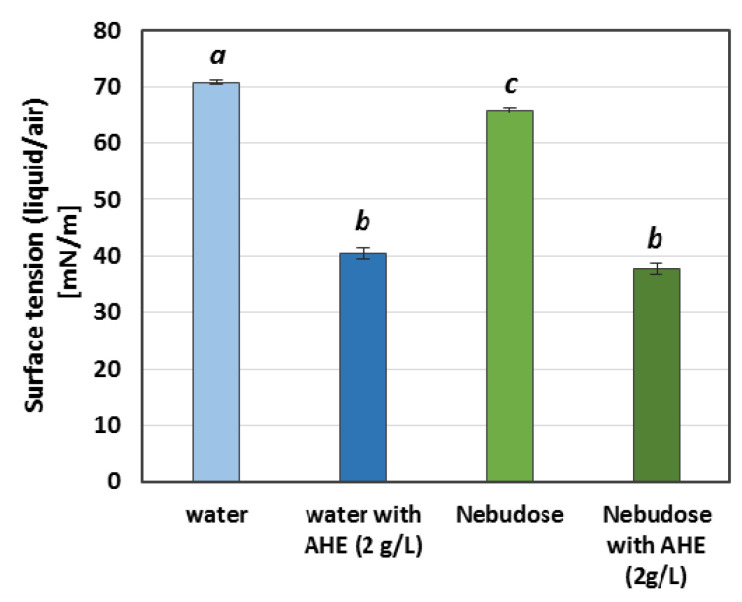
The surface tension of the emulsion components. Small, italicized letters in the same column indicate the statistical similarities and differences between the data series (*p* < 0.05).

The emulsion samples were prepared, and a stability test was performed for each sample. Investigations of the emulsification index (EI) were conducted 24 h and 7 days after the preparation of the emulsion systems. Simultaneously, the samples were visually observed with the naked eye. The results presented in Table 2 show significant differences in EI between the samples with different liquid phases, which were water or Nebudose. While after both 24 h and 7 days the level of emulsification in the samples prepared with water was close to 100%, the EI values did not exceed 10% in the samples with a Nebudose liquid phase. As the differences in surface tension between water and Nebudose were relatively small (Figure 1), it can be assumed that the decisive influence on such a decrease in the emulsion stability in the samples containing Nebudose was a result of the NaCl (0.9%) and hyaluronic acid (0.1%) contents in the solution causing a salting-out effect. In that case, the addition of the extract of *A. hippocastanum* as an emulsifier would provide an advantage. While this extract did not significantly change the value of the emulsification index in the samples with water (although a slight decrease in this parameter was observed), the level of emulsion content was significantly higher in the systems with Nebudose and AHE. It was clearly visible (with the naked eye) when the concentration of the extract exceeded 1 g/L. This value surpassed the critical concentration of extract’s micellisation [21], so it can be assumed that, in a system saturated with surfactants, the stabilization of the oil droplets can be improved by the presence of a layer of saponins on the outer surface of the drop. This promotes a reduction in surface tension, as illustrated in Figure 1.

**Figure 2:**
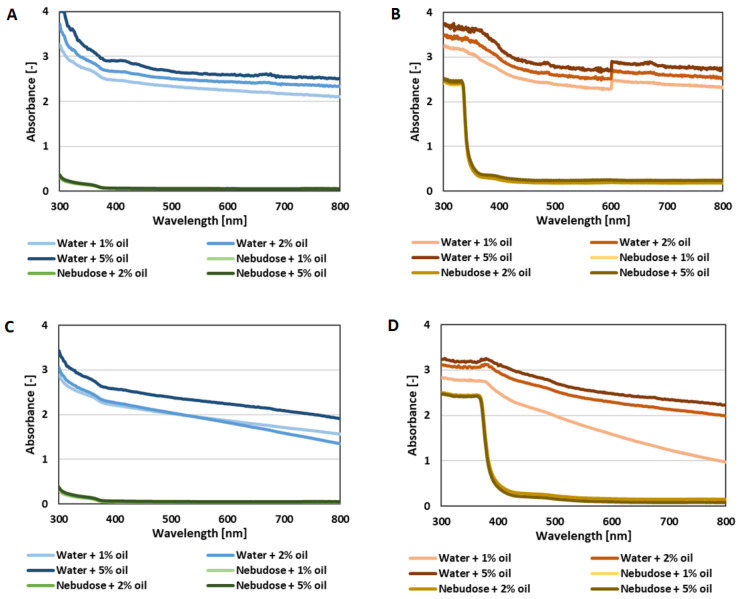
Visible light spectra of the emulsions after 24 h (**A**,**B**) and after centrifuging at 4500 rpm for 10 min (**C**,**D**); the samples without AHE (**A**,**C**) and with 2 g/L AHE (**B**,**D**).

Further information was obtained after the analysis of the visible light spectra of the emulsions, which are presented in Figure 2. The absorbance of the colloidal systems at wavelengths in the range of UV–Vis corresponded to the particle/droplet distribution and size [23]. Nevertheless, it is justified to analyse the whole spectrum for a wider perspective, which would help to avoid the potential interferences with other suspension components. In this study, a strong signal was observed for AHE-containing samples, especially at wavelengths below 360 nm. Samples with AHE were also observed to show an upward shift in all spectra. Hence, absorbance measurements at 600 nm were concluded to be misleading, and were therefore excluded from our study. In samples without AHE, the increase of absorbance below 400 nm can be explained by the absorbance of hemp seed oil components such as chlorophyll. The same behaviour was observed in our previous study [24]. Nevertheless, the spectra of the water samples indicated stronger absorbance, confirming the higher dispersion of the oil droplets compared to Nebudose-based emulsions. As previously mentioned, the spectra of the emulsions containing 2 g/L AHE showed strong absorbance below 360 nm, which can be explained by the extract components [21]. However, even for the samples with 2 g/L AHE, a stronger absorbance of water samples was noticeable. Moreover, a decrease of the absorbance at higher wavelengths was observed when the samples were centrifuged. This could be a result of the higher tendency of larger droplets to aggregate and coalescence. This might lead to lower light dispersion, especially in higher wavelengths.

### 2.2. Droplet Size Determination

The stability test showed that, in samples with 0.5 g/L and 1.0 g/L, the addition of AHE resulted in the lowest stability. In Table 3, we present the average values from the tripled measurements of the emulsion droplet size d-ave (intensity-based overall average size, determined by the cumulative method) with main peaks maxima and PDI indexes. The obtained results of the d-ave highlighted that the liquid phase changes had a strong effect on the emulsion droplet size. A more intensive impact on the AHE concentration was observed in Nebudose emulsions. At the lowest oil concentration (1%), the emulsion systems without AHE contained larger particles (average d-ave value) than the emulsion with AHE. In addition, the PDI indexes were relatively smaller for the samples in which AHE was used as the stabilizer and water as the liquid phase.

**Figure 3:**
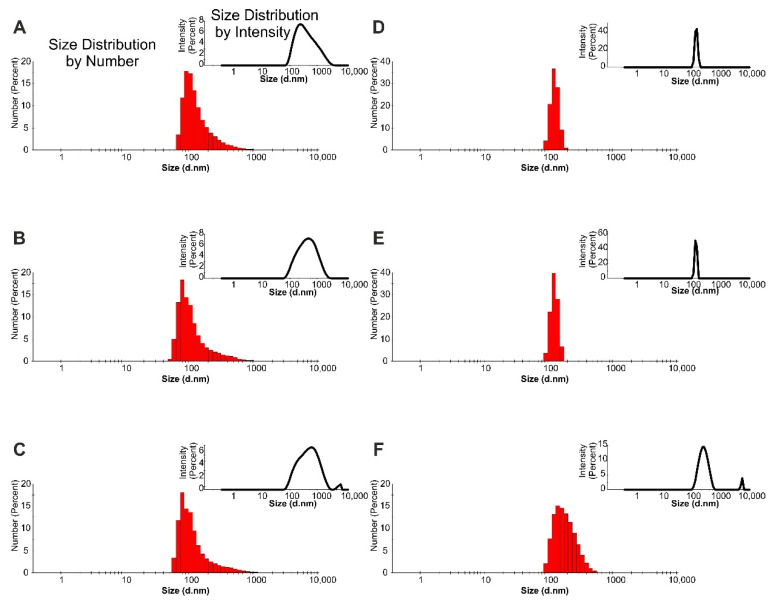
Droplet size distribution of the samples containing AHE (2.0 g/L) obtained with DLS measurements: (**A**–**C**) water solutions with 1, 2, 5% HSO content (respectively); (**D**–**F**) Nebudose solution with 1, 2, 5% HSO content (respectively).

Based on our previous studies [25,26], we decided to present the droplet size distribution by intensity and number for the samples containing AHE (Figure 3). Especially in the highly-polydispersed samples, such as the prepared emulsions (some of PDI values archived 1.0), the intensity of the scattered light from even a few large particles (possible contamination, aggregates, agglomerates, larger droplets) strongly affected the average particles’ diameter. One of the frequently-used approaches for the elimination of this phenomenon is the use of a filtration process, but then there are no ‘real conditions’ in the investigated samples. Comparing the results of particle size vs. number for the emulsions with AHE, smaller particles were recorded in the water-based systems than in the Nebudose. Furthermore, as we predicted, the signal from larger particles had a strong impact on the result of particle size vs. intensity (see Figure 3A–C). Some unexpected results were recorded for the Nebudose samples, which showed high PDI values (0.441–1.000). The d-ave values were a few microns for 1 and 2% of the HSO content emulsion, but the peaks maxima were recorded at 148 and 124 nm, respectively. Some explanation brings the raw correlation data obtained during DLS measurements. The shape of the autocorrelation curve (data not shown) suggested that more than one particle fraction occurred in the investigated system; it corresponds with a PDI value of 1.000. Therefore, we decided to perform additional microscopic tests (see p. 2.5.) other than DLS [27] in order to evaluate the emulsion droplet/particle size.

**Figure 4:**
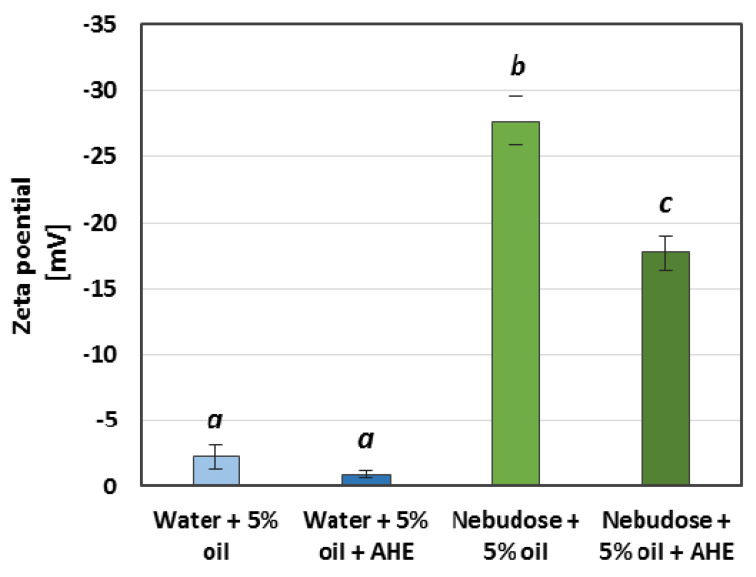
Zeta potential values of the tested emulsions’ droplets. AHE was applied in the final concentration of 2 g/L. Small italicized letters indicate the statistical differences between the data series.

A further part of the research was devoted to the analysis of zeta potential. These supplementary measurements are only presented for the selected samples (Figure 4). The saponin-rich AHE led to an increase of the zeta potential in the tested systems. For the water emulsions, it was a slight rise from −2.25 mV to −0.95 mV; however, in the case of the Nebudose emulsions, the increase of the zeta potential was more significant (from −27.74 mV up to −17.72 mV). Hence, the zeta potential changes can be correlated with the addition of the natural surfactants present in AHE. The relatively lower zeta potential value for the sample with a Nebudose liquid phase without AHE could be caused by the ions (i.e., Na^+^ and Cl^−^) or other compounds which contain this commercially-available solution. We can conclude that, for the emulsion stability in which saponin surfactants were used as a stabilizers, the DLS and zeta potential measurements should be verified by additional stability tests, including visual studies.

Our obtained zeta potential results can be explained by some previous studies in which saponins were used as a stabilizer for vegetable oil emulsions. Da Faria et al. [28] proved the saponin-emulsifying properties of β-lactoglobulin and *Quillaja* bark. They also observed that the zeta potential magnitude significantly decreased with increased salt concentrations, which agrees with our research, in which a lower potential in Nebudose emulsions in contrast to water emulsions was observed due to higher ion concentrations. Perichapan et al. [29] observed that *Quillaja saponaria* saponins enhanced the emulsion creation of rice bran oil, with a simultaneous reduction of the emulsion droplet size. In contrast to our study, they observed only a slight decrease of the droplets’ zeta potential when saponins were used. A strong decrease of the droplets’ zeta potential was noticed for soya saponins by Zhu et al. [30], in which corn oil was emulsified. However, the AHE saponins and other components possess molecular structures that are different from *Quillaja saponins*. Their high hydroxyl group content, which was confirmed in a previous study [21], may lead to a higher polarity gradient near the droplet’s surface, which increases the ion concentration, as was discussed above.

### 2.3. Rheological Properties

**Figure 5:**
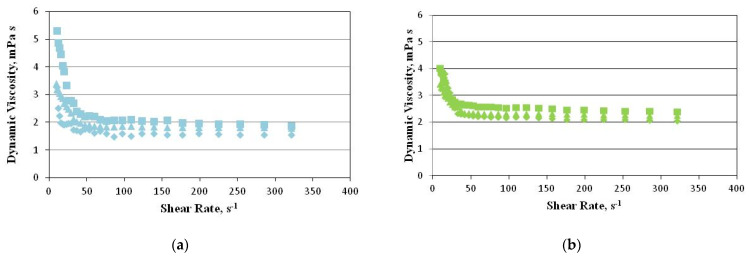

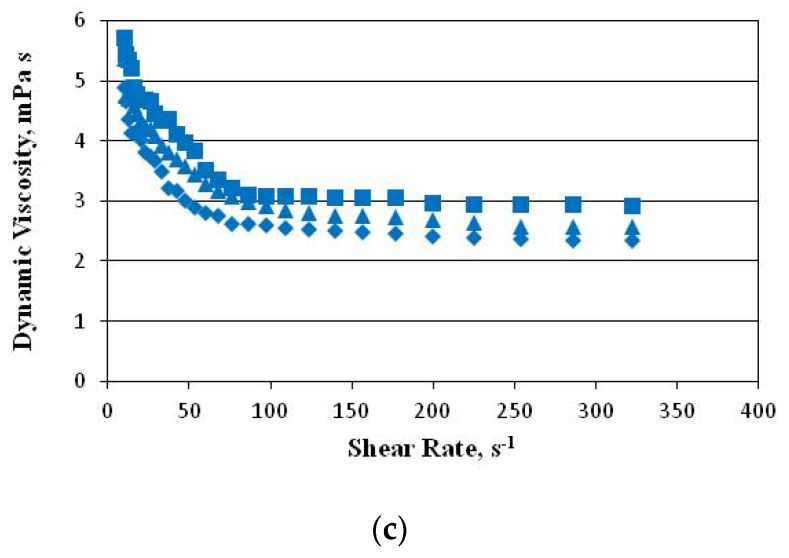
The dependence between the dynamic viscosity and the shear rate of the model O/W emulsions: (**a**) HSO in water; (**b**) HSO in Nebudose; (**c**) HSO in water with AHE. ◆—1%; ■—2%; ▲—5%.

For detailed rheological measurements, we took only the samples of which the phases didn’t separate so quickly (we excluded samples 2 and 2% HSO with AHE in Nebudose). Figure 5 shows the relationship between the dynamic viscosity and the shear rate of the emulsions at a temperature of 24 °C on the day when they were prepared. The samples with water or Nebudose with AHE at a concentration of 2 g/L were used.

The power model (1) was used to describe the viscosity curves:

(1) $$\eta =K{\left(\overset{\cdot }{\gamma }\right)}^{n-1}$$

in which *η* is the dynamic viscosity, *K* is the consistency coefficient, $\overset{\cdot }{\gamma }$ is the shear rate, and *n* is the flow index.

Table 4 shows the parameters of the power model describing the viscosity curves of the model emulsion systems with different concentrations of hemp seed oil. A sample containing 5% hemp seed oil was used for the tests due to the low stability of emulsions containing a saponin-rich extract and Nebudose. The high values of the coefficient of determination (*R*^2^ = 0.89 − 0.97) indicate that the applied model perfectly describes the flow of the emulsions within the applied range of the shear rate. The flow index (n) values (Table 4) of all of the emulsion systems were less than one, which points to a non-Newtonian shear-thinning flow.

The value of the consistency coefficient (K), which is a measure of the initial system viscosity, tended to increase along with the concentration of hemp seed oil in the emulsion.

The increase in the shear rate reduced the dynamic viscosity, and showed two zones depending on the value of this parameter. The dynamic viscosity dropped sharply at low shear rates, in which—as at higher rates—the changes in the viscosity were minimal. These differences between the emulsions were more pronounced at low shear rates, in which the forces between the particles were dominant, rather than the hydrodynamic forces imposed by the shear flow. As the shear rate increased, the hydrodynamic influence prevailed, and finally the shear viscosity curves converged. This indicates a continuous breakdown of the structure of the emulsion aggregates, which results in a lower flow resistance [31]. This is parallel with the observations made by Rave et al. (2020) [32] in their study on O/W emulsions, by Zheng (2019) [33] in their study on semi-solid foods, by Zhu et al. (2019) [34] in their study on Bengal gram flour suspensions, as well as by Zhu et al. (2019) and Domian and Szczepaniak (2020) [34,35], who studied concentrated food emulsions.

The increase in the emulsion viscosity accompanied by the increase in the amount of oil was caused by molecular movements, and the formation of interphase layers and physical barriers with the emulsion components. After seven days of storage, the emulsions under analysis (the results have not been presented) were characterized by a similar relationship between their dynamic viscosity and shear rate.

**Figure 6:**
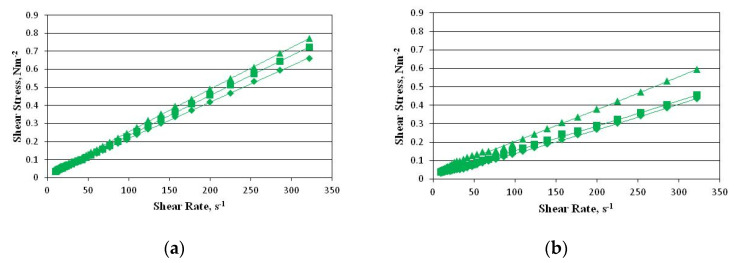
Examples of the flow curves of the model emulsions of HSO in water with Nebudose after 1 (**a**) and 7 (**b**) days: ◆—1%; ■—2%; ▲—5%.

Figure 6 shows examples of the flow curves of model emulsions (the shear stress vs. the shear rate).

The flow curves provide information about the behaviour and nature of the rheological properties of the samples. In the emulsions under analysis, this behaviour was typical of a non-Newtonian pseudoplastic fluid. As Table 5 shows, the rheological properties of the samples were in strict accordance with the Herschel–Bulkley model (*R*^2^ = 0.90–0.99), which is described by Equation (2) below:

(2) $$\tau =\tau_{0}+K{\left(\overset{\cdot }{\gamma }\right)}^{n}$$

in which *τ*_0_ is the shear stress, *K* is the consistency coefficient, $\overset{\cdot }{\gamma }$ is the shear rate, and *n* is the flow index.

The presence of the yield point proves that, below the value of the shear stress corresponding to the yield point, the emulsion components form three-dimensional structures. In which, as the low value of the yield point indicates, the analyzed emulsions in the no-flow conditions are poorly cross-linked systems.

The value of the yield point tended to increase along with the oil concentration. Generally, the value of the yield point is determined by the volume fraction of the particles, a decrease in the size of the particles, and an increase in the intermolecular forces [35]. As the sizes of the particles in all of the emulsions are comparable, it is possible to relate the yield point value to the increase in the volume of the dispersed phase, and thus to the increase in the repulsive forces between adjacent droplets.

The consistency coefficient *K* is a rheological parameter which depends on the forces of attraction between the fluid particles, and accordingly on the type and size of the particles in the dispersed phase, as well as on the temperature. The consistency coefficient *K* is related to the viscosity, and it determines the resistance of the fluid to deformation depending on the shear force. Consequently, the value of this parameter tended to increase along with the content of the oil in the emulsions under analysis.

The stability of all of the emulsions deteriorated after their storage for 7 days at room temperature. Consequently, their viscosity decreased significantly. The goodness-of-fit parameters of the Herschel–Bulkley model had lower values. However, the trend of the changes in their values was the same as before their storage. The decrease in the viscosity of the emulsions during storage may have been caused by two factors: the diffusion of water molecules from the inner to the outer aqueous phase, or their coalescence.

### 2.4. FTIR Studies of the Emulsion Systems

**Figure 7:**
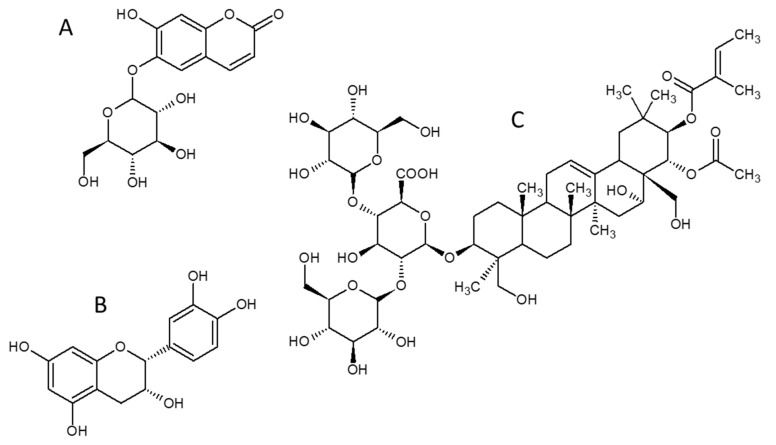

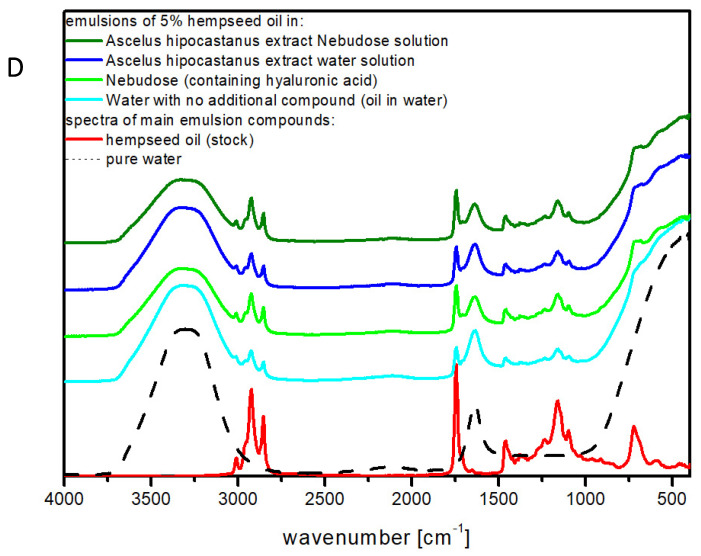
Molecular structures of the selected AHE main components: (**A**) esculin, (**B**) β-aescin, (**C**) (-)-epicatechin [38,39]; (**D**) FTIR spectra of water–oil emulsions for 5% of oil content (24 h after sonication).

Based on the preliminary tests, we selected the most stable samples containing at least 2 g/L AHE and 5% hemp seed oil contents. Considering the high demand for omega-3 and omega-6 acids present in emulsion formulations for both cosmetic and nutritional applications, we decided to examine the possible changes in the structure or oil components ratio as a result of the emulsification, by FTIR methods. Figure 7 presents the FTIR spectra of O/W emulsions prepared and stabilized using different surfactant components (each additional agent can be considered as a surfactant component). The FTIR spectrum of the hemp seed oil used is presented in red in Figure 7. It is a typical oil spectrum with a high content of fatty compounds of both edible and fossil oils [36,37]. The high relative intensity of the 1745 cm^−1^ band (characteristic for C = C bond) suggests a very high content of unsaturated fatty acids. Nevertheless, the observed signals may come from the main components of AHE, including esculin, β-aescin or (-)-epicatechin [38,39] (Figure 7). Figure 7 demonstrates that the emulsions consisting of only water and oil contained the lowest amount of hemp seed oil components, with no change in their ratios. The use of Nebudose or AHE led to the incorporation of higher amounts of hemp seed oil components into the emulsion. In the emulsions, Nebudose provided the efficient integration of unsaturated carbon chains, despite its low stabilizing ability. This suggests that the last emulsion formulation is the most promising agent for further applications.

### 2.5. Microscopic Analysis

**Figure 8:**
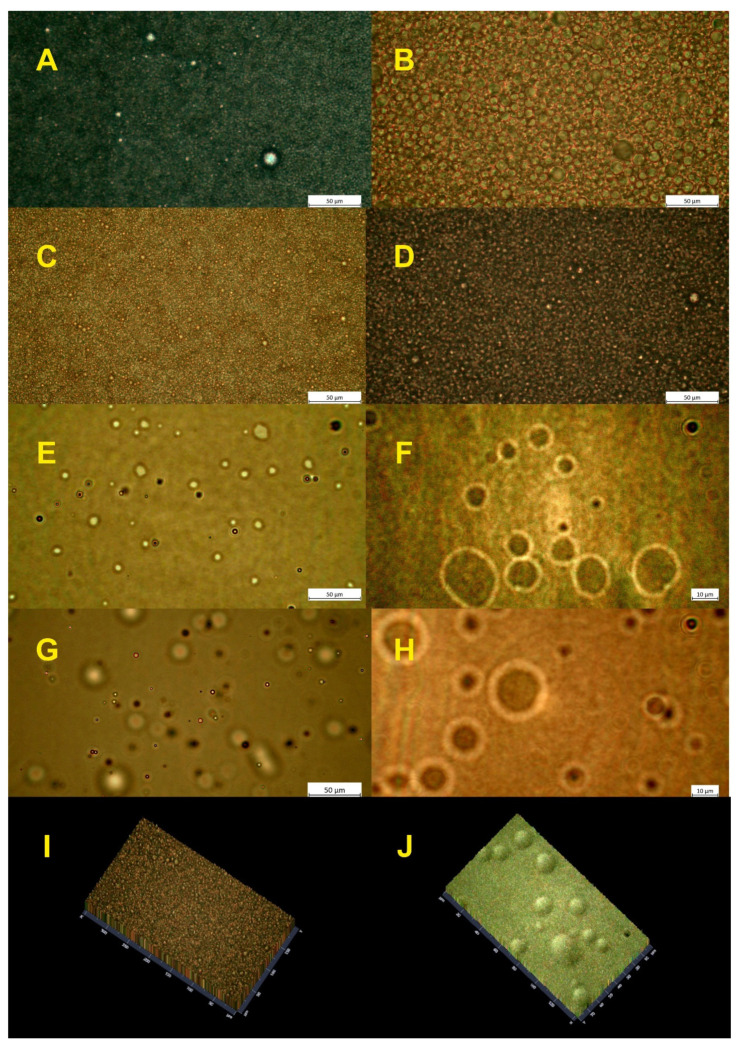
Light microscopy images of 5% HSO in water emulsions: without AHE (**A**) and (**B**) (magnification ×40 and ×100, respectively); with 2 g/L AHE (**C**) and (**D**) (magnification ×40 and ×100 respectively); 5% HSO in Nebudose emulsions: without AHE (**E**) and (**F**) (magnification ×40 and ×100 respectively); with 2 g/L AHE (**G**) and (**H**) (magnification ×40 and ×100 respectively); (**I**) 2.5 D images of the 5% HSO in water emulsion with 2 g/L AHE; (**J**) 2.5 D images of 5% HSO in Nebudose emulsion with 2 g/L.

A reversed microscope and a special cuvette with microchannels were used to verify the droplet size and homogeneity of the samples. Figure 8 presents the comparison between the most stable samples containing 5% HSO in water (Figure 8A–D) and Nebudose (Figure 8E–H) liquid phases. The observations were made using different magnifications: ×40 (Figure 8A,C,E,G) and ×100 (Figure 8B,D,F,H). The microscopic analysis supports the results obtained by the DLS particle size distribution and naked eye observations. It was observed that samples with water as the liquid phase stabilized by saponin reached the desired homogeneity and a smaller particle size of plant extract surfactants. Visible differences of droplet size in the emulsion systems without AHE and with AHE (Figure 8B and Figure 8D, respectively) were recorded. The samples with water as the liquid phase had a better homogeneity than the Nebudose emulsions (Figure 8E–H). The droplet size in the Nebudose emulsions varied greatly, and large droplets were observed to be present. The presence of larger droplets may be caused by a faster coalescence (see Figure 8F,H, in which a higher software magnification was used) and/or emulsion destabilization. The microscopic analysis confirmed the results obtained by the DLS of the Nebudose emulsions, in which droplets of dozens of nm in size and droplets in the range of few micrometers were recorded. The microscopic imaging further proved that the high PDI index of the Nebudose emulsion systems was caused by the high inhomogeneity of the samples. Lastly, we presented 2.5 D images to exhibit the differences in the structure of the water-based and Nebudose-based emulsions (see Figure 8I and Figure 8J, respectively). In the Nebudose emulsions, even at the larger magnification, only a few droplets were observed, with a varying distances between them. The opposite observation was made for the HSO in water emulsion, in which the droplets appeared side-by-side and were uniformly dispersed. For detailed studies of the droplet size and size distribution, we recommend the use of a confocal laser scanning microscope or a scanning electron microscope with cryo-mode.

## 3. Materials and Methods

### 3.1. Reagents

For all of the experiments, chemicals of analytical grade were used. The solvents and reagents were purchased from Sigma-Aldrich (Poland). The hemp seed oil (HSO) and Nebudose (0.9% NaCl and 0.1% hyaluronic acid water solution) were purchased from Złoto Polskie (Poland) and Solinea (Poland), respectively. The plant material, *Aesculus hippocastanum* L. bark, was obtained from Flos (Poland), and was used to prepare a saponins-rich extract, as described by Jarzębski et al. [21]. Briefly, 10 g dry AHE bark was extracted for 6 h with 100 mL methanol in a Soxhlet apparatus after solvent evaporation; the extract was freeze-dried and kept in 4 °C. Deionized water (18.2 MΩ cm^−1^) was prepared using the Arium pro DI water deionization system (Sartorium Stedim, Poland).

### 3.2. Emulsion Preparation

Emulsion samples of 20 mL were prepared in sterile 50 mL plastic laboratory tubes. The samples were first mixed (Vortex 3, IKA, Poland) at 2500 rpm for 15 s, and then homogenized (sonicator Sonoplus, Bandelin, Berlin, Germany) in the following conditions: 10 min, in cycles action/break 10 s/10 s, amplitude 16%, and cooled with tap water. The hemp seed oil content in the samples was 1%, 2%, or 5% *v/v*. Deionized water or Nebudose were applied as the non-oil phase. In samples with an emulsifier, *A. hippocastanum* extract (AHE) was added in order to obtain a concentration of 2 gL^−1^. The procedure was conducted in a laminar chamber (Class II, E2 Type, Esco, Singapure) after UV sterilization, which provided an aseptic work environment. The samples were transferred into 10 mL plastic tubes and stored in darkness at 22 ± 1 °C for further analysis.

### 3.3. Methods

#### 3.3.1. Stability Tests

The experiment was conducted according to Camacho-Chab, J.C. et al. (2013) [40]. The emulsion stability tests were conducted after 24 h and 7 days, and the emulsification indices (E_24_ and E_7d_) were calculated. The emulsifying activity was determined as the ratio of the height of the emulsified layer to the total height that was occupied by the emulsion after a defined time period.

#### 3.3.2. Surface Tension

The surface tension of the HSO and solutions with non-oil phases were measured by the du Nuoy platinum ring method using a K20 tensiometer (Krüss, Hamburg, Germany). All of the measurements were conducted at 22 ± 1 °C.

#### 3.3.3. UV-Vis Test

For the spectrophotometric analysis (after 24 h), 200 μL of each emulsion sample was taken from the middle of the height of the test tubes and analyzed on a wide range of wavelengths between 300 and 900 nm (Multiskan, Thermo Scientific, Dreieich, Germany). The remaining part of the 10 mL sample was centrifuged for 10 min, reaching an angular velocity of 4500 g, and then analyzed as described before. The remaining 10 mL sample was subjected to exactly the same treatment after the seventh day of its storage.

#### 3.3.4. DLS and Zeta Potential

The Zetasizer Nano-ZS (Malvern, Malvern, United Kingdom) was applied to measure the hydrodynamic diameters (d_H_) of the particles and the zeta potential (ζ) of the emulsion samples. The DLS autocorrelation functions were registered from the scattered light, which was recorded at an angle of 173°. The particle size distribution and zeta potential measurements were performed with the automatic settings mode at 25 °C. Immediately prior to the examinations, the samples were thermostated at 25 °C for 5 min inside the measurement cell in order to obtain the zeta potential measurements. The measurement series values and their respective standard deviations were obtained from an average of five measurements.

#### 3.3.5. Viscosity

The rheological properties of the emulsions were determined by means of a programmable rotational ViscoQC 300 viscometer (Anton Paar Gmbh, Graz, Austria). The tests were conducted at 25 ± 0.5 °C, using the ‘double-gap’ DG26 system with an L1 spindle. The speed of the spindle ranged from 7 to 250 rpm, with an increasing trend, which corresponds to a shear rate of 10 to 322 1/s. Each shear rate was imposed for 1 min in order to stabilize the viscosity. Each test was performed in triplicate, with fresh samples.

#### 3.3.6. FTIR

The FTIR spectra were obtained using a Spectrum Two FT-IR Spectrometer equipped with a Universal ATR with a diamond crystal (PerkinElmer, Waltham, MA, USA). The data were collected over a spectral range of 400–500 cm^−1^.

#### 3.3.7. Microscopic Investigations

The microscopic investigations were performed using an inverted microscope ZEISS Axio Vert.A1 (Zeiss, Shanghai, China). The studies were performed with the objective magnifications LD A-Planx40/0,55 ph1 (air), A-Planx100/1,25 Oil Ph2 oil, and the color camera Axiocam 208 (Zeiss, Shanghai, China). For the imaging, the emulsions were inserted into a 1 μ-Slide VI^0,1^ cuvette (ibidi GmbH, Gräfelfing, Germany). For the presentation, the resolution of the images were automatically adjusted by the best fit ZEN2.5 software (Zeiss, Jena, Germany).

#### 3.3.8. Statistical Analysis

All of the experiments were conducted in three independent repetitions. The mean values and standard deviations were presented in the manuscript. The statistical significance of the differences between the series of results were confirmed by an analysis of variance. The calculations and graphical presentation of the results were made with Microsoft Office Excel 2013 (Microsoft, Redmond, WA, USA).

## 4. Conclusions

Plant extracts containing saponins are promising natural surfactants to be used as nanoemulsion stabilizers. It can be concluded that the higher HSO content (5%) and the highest tested AHE concentration (2 g/L) promoted the stability of the emulsions. The DLS measurements also confirmed the smallest droplet size for the samples with AHE. However, the experimental results showed that the addition of other components, such as hyaluronic acid in an NaCl solution, needs further detailed studies, which we aim to explore in the next steps. Furthermore, in samples with saponin-rich plant extract, determination of droplet size (by DLS) and zeta potential were not sufficient in defining their behaviour. Therefore, we strongly recommend the use of various microscopic techniques for detailed studies of the emulsion structure. As we presented here, even a visible-light microscope might be applied for a general emulsion homogeneity evaluation. On the other hand, based on our previous research, in which the antimicrobial activity of the horse chestnut was proved, the composition of AHE and HA might have beneficial effects on the development of new biomedical and food formulations. The combination of AHE and HA with hemp seed oil in emulsion systems can, overall, increase its nutritional benefits, making them great candidates in food formulations.

## Figures and Tables

**Table 1:** Content of the prepared emulsion samples.

Sample No.	Hemp Seed Oil Content (%)	Liquid Phase	AHE Concentration(g/L)
1	1	water	0
2			0.5
3			1.0
4			2.0
5	2	water	0
6			0.5
7			1.0
8			2.0
9	5	water	0
10			0.5
11			1.0
12			2.0
13	1	Nebudose	0
14			0.5
15			1.0
16			2.0
17	2	Nebudose	0
18			0.5
19			1.0
20			2.0
21	5	Nebudose	0
22			0.5
23			1.0
24			2.0

**Table 2:** The emulsification index of the tested samples after 24 h and 7 days.

Liquid Phase	HSO Content (%)	AHE Concentration(g/L)	Emulsification Index [%]	
			24 h	7 days
water	1	0	99 ± 1 (*a*)	98 ± 1 (*a*)
		0.5	99 ± 1 (*a*)	96 ± 2 (*ab*)
		1.0	99 ± 1 (*a*)	96 ± 3 (*ab*)
		2.0	99 ± 1 (*a*)	95 ± 3 (*ab*)
	2	0	98 ± 2 (*a*)	98 ± 2 (*ab*)
		0.5	96 ± 2 (*ab*)	93 ± 2 (*b*)
		1.0	98 ± 2 (*ab*)	95 ± 2 (*b*)
		2.0	96 ± 3 (*ab*)	96 ± 3 (*ab*)
	5	0	98 ± 2 (*ab*)	98 ± 2 (*ab*)
		0.5	98 ± 1 (*ab*)	95 ± 2 (*b*)
		1.0	96 ± 2 (*ab*)	93 ± 3 (*b*)
		2.0	94 ± 4 (*ab*)	94 ± 4 (*b*)
Nebudose	1	0	1 ± 1 (*c*)	0 ± 0 (*e*)
		0.5	2 ± 1 (*c*)	0 ± 0 (*e*)
		1.0	3 ± 2 (*cd*)	1 ± 1 (*cd*)
		2.0	6 ± 2 (*d*)	5 ± 1 (*d*)
	2	0	1 ± 1 (*c*)	0 ± 0 (*e*)
		0.5	2 ± 1 (*c*)	0 ± 0 (*e*)
		1.0	3 ± 1 (*cd*)	1 ± 1 (*c*)
		2.0	6 ± 2 (*d*)	5 ± 1 (*d*)
	5	0	1 ± 1 (*c*)	0 ± 0 (*e*)
		0.5	3 ± 2 (*cd*)	0 ± 0 (*e*)
		1.0	5 ± 2 (*d*)	2 ± 1 (*cd*)
		2.0	8 ± 3 (*d*)	6 ± 1 (*d*)

All values for Emulsification Index are means of triplicate ± SD. Small, italicized letters in the same column indicate the statistical similarities and differences between the data series *(p <* 0.05).

**Table 3:** Emulsion droplet size values (d-ave), PDI and average peaks maxima.

Hemp Seed Oil Content (%)	Liquid Phase	AHE Conc. (g/L)	d-ave (nm)	PDI	1st Peak Max (nm)	2nd Peak Max (nm)
1	water	0	296 ± 3 *(a)*	0.398 ± 0.035 *(a)*	459 ± 47 *(a)*	2935 ± 2574 *(a)*
		2.0	275 ± 1 *(b)*	0.283 ± 0.010 *(b)*	452 ± 37 *(a)*	81 ± 47 *(b)*
2		0	357 ± 8 *(c)*	0.434 ± 0.010 *(c)*	661 ± 62 *(a)*	32 ± 28 *(bc)*
		2.0	298 ± 4 *(a)*	0.306 ± 0.045 *(d)*	445 ± 33 *(a)*	35 ± 20 *(bc)*
5		0	393 ± 8 *(d)*	0.516 ± 0.008 *(e)*	750 ± 89 *(a)*	3427 ± 2968 *(a)*
		2.0	306 ± 5 *(e)*	0.468 ± 0.033 *(c)*	570 ± 86 *(a)*	2783 ± 1703 *(a)*
1	Nebudose	0	1527 ± 409 *(f)*	0.812 ± 0.174 *(f)*	1087 ± 222 *(a)*	98 ± 42 *(b)*
		2.0	2533 ± 923 *(g)*	1.000 *(g)*	148 ± 19 *(a)*	10 ± 16 *(c)*
2		0	2099 ± 483 *(g)*	0.601 ± 0.192 *(e)*	843 ± 219 *(a)*	47 ± 27 *(bc)*
		2.0	1512 ± 239 *(f)*	1.000 *(g)*	124 ± 21 *(a)*	---
5		0	4301 ± 314 *(h)*	0.442 ± 0.009 *(c)*	2758 ± 1418 *(a)*	---
		2.0	283 ± 10 *(a)*	0.606 ± 0.112 *(e)*	313 ± 104 *(a)*	3569 ± 2976 *(a)*

All values are means of triplicate ± SD. Small italicized, letters indicate the statistical similarities and differences between the data series (*p* < 0.05).

**Table 4:** Average value and SD of the parameters of the power model describing the viscosity curves of the O/W emulsions: consistency coefficient (K), flow index (n).

HSO Content (%)	Liquid Phase	AHE Concentration (g/L)	24 h		7 days	
			K (Pa·s)	n	K (Pa·s)	n
1	water	0	0.0053 ± 0.0004 *(a)*	0.79 ± 0.05 *(a)*	0.0037 ± 0.0004 *(c)*	0.70 ± 0.05 *(a)*
		2.0	0.0079 ± 0.0006 *(b)*	0.76 ± 0.05 *(ab)*	0.0070 ± 0.0003 *(ab)*	0.75 ± 0.05 *(a)*
2		0	0.0059 ± 0.0004 *(a)*	0.78 ± 0.05 *(a)*	0.0045 ± 0.0003 *(d)*	0.71 ± 0.05 *(ab)*
		2.0	0.0084 ± 0.0007 *(b)*	0.78 ± 0.05 *(a)*	0.0079 ± 0.0004 *(b)*	0.74 ± 0.05 *(a)*
5		0	0.0064 ± 0.0006 *(ae)*	0.68 ± 0.04 *(b)*	0.0056 ± 0.0006 *(a)*	0.62 ± 0.04 *(b)*
		2.0	0.0092 ± 0.0008 *(b)*	0.79 ± 0.05 *(a)*	0.0087 ± 0.0005 *(b)*	0.78 ± 0.05 *(a)*
1	Nebudose	0	0.0054 ± 0.0005 *(a)*	0.77 ± 0.03 *(a)*	0.0044 ± 0.0005 *(cd)*	0.72 ± 0.05 *(ab)*
		2.0	---	---	---	---
2		0	0.0061 ± 0.0005 *(a)*	0.83 ± 0.06 *(a)*	0.0057 ± 0.0002 *(a)*	0.81 ± 0.06 *(a)*
		2.0	---	---	---	---
5		0	0.0070 ± 0.0006 *(ab)*	0.85 ± 0.07 *(a)*	0.0068 ± 0.0003 *(e)*	0.80 ± 0.06 *(a)*
		2.0	0.0093 ± 0.0008 *(b)*	0.80 ±0.06 *(a)*	0.0089± 0.0005 *(b)*	0.80 ± 0.05 *(a)*

Small, italicized letters indicate the statistical differences between the data series (*p* < 0.05).

**Table 5:** Average value and SD of the parameters of the Herschel–Bulkley model describing the flow curves of the O/W emulsions: shear stress (*τ*_0_) consistency coefficient (K), flow index (n).

HSO Content (%)	Liquid Phase	AHE Concentration (g/L)	24 h			7 days		
			τ_o_, (Pa)	K (Pa·s)	n	τ_o_, (Pa)	K, (Pa·s)	n
1	water	0	0.018 ± 0.004 *(ag)*	0.0025 ± 0.0001 *(a)*	0.89 ± 0.09 *(a)*	0.011 ± 0.006 *(g)*	0.0016 ± 0.0001 *(f)*	0.73 ± 0.05 *(b)*
		2.0	0.050 ± 0.001 *(b)*	0.0038 ± 0.0001 *(b)*	0.94 ± 0.05 *(a)*	0.032 ± 0.005 *(a)*	0.032 ± 0.005 *(b)*	0.79 ± 0.03 *(b)*
2		0	0.042 ± 0.004 *(c)*	0.0027 ± 0.0002 *(a)*	0.89 ± 0.16 *(a)*	0.021 ± 0.006 *(ag)*	0.0018 ± 0.0001 *(g)*	0.89 ± 0.05 *(a)*
		2.0	0.066 ±0.002 *(d)*	0.0041 ± 0.0002 *(b)*	0.94 ± 0.06 *(a)*	0.049 ± 0.006 *(c)*	0.0033 ± 0.0003 *(b)*	0.75 ± 0.18 *(b)*
5		0	0.050 ±0.001 *(b)*	0.0029 ± 0.0003 *(a)*	0.89 ± 0.21 *(a)*	0.031 ± 0.001 *(a)*	0.0023 ± 0.0001 *(h)*	0.79 ± 0.07 *(ab)*
		2.0	0.070 ± 0.001 *(e)*	0.0046 ± 0.0002 *(c)*	0.96 ± 0.09 *(a)*	0.052 ± 0.003 *(b)*	0.0040 ±0.0008 *(bc)*	0.68 ± 0.08 *(bc)*
1	Nebudose	0	0.021 ± 0.005 *(ag)*	0.0030 ± 0.0001 *(a)*	0.94 ± 0.02 *(a)*	0.010 ± 0.008 *(g)*	0.0020 ± 0.0001 *(g)*	0.59 ± 0.05 *(c)*
		2.0	---	---	---	---	---	---
2		0	0.027 ± 0.005 *(a)*	0.0032 ± 0.0001 *(a)*	0.94 ± 0.05 *(a)*	0.024 ± 0.008 *(ag)*	0.0022 ± 0.0001 *(g)*	0.67 ± 0.05 *(bc)*
		2.0	---	---	---	---	---	---
5		0	0.046 ± 0.009 *(b)*	0.0035 ± 0.0002 *(d)*	0.94 ± 0.09 *(a)*	0.049 ± 0.003 *(b)*	0.0025 ± 0.0002 *(g)*	0.69 ± 0.16 *(bc)*
		2.0	0.076 ± 0.003 *(f)*	0.0050 ± 0.0002 *(e)*	0.97 ± 0.07 *(a)*	0.053 ± 0.003 *(b)*	0.0042 ± 0.0005 *(b)*	0.69 ± 0.08 *(bc)*

Small italicized, letters in the same column indicate the statistical differences between the data series (*p* < 0.05).

## Data Availability

The raw/processed data required to reproduce these findings cannot be shared at this time as the data also forms part of an ongoing study.
